# Changes in Watering Frequency Stimulate Differentiated Adaptive Responses among Seedlings of Different Beech Populations

**DOI:** 10.3390/biology11020306

**Published:** 2022-02-14

**Authors:** Georgios Varsamis, George C. Adamidis, Theodora Merou, Ioannis Takos, Katerina Tseniklidou, Panayiotis G. Dimitrakopoulos, Aristotelis C. Papageorgiou

**Affiliations:** 1 Department of Forest and Natural Environment Sciences, International Hellenic University, 66100 Drama, Greece; thmerou@for.ihu.gr (T.M.); itakos@for.ihu.gr (I.T.); katerina.tseniklidou@gmail.com (K.T.); 2 Section of Plant Biology, Department of Biology, University of Patras, Rio, 26504 Patras, Greece; adamidis@upatras.gr; 3 Biodiversity Conservation Laboratory, Department of Environment, University of the Aegean, 81100 Mytilene, Greece; pdimi@env.aegean.gr; 4 Department of Molecular Biology and Genetics, Democritus University of Thrace, Dragana, 68100 Alexandroupolis, Greece

**Keywords:** beech, stem anatomy, adaptive traits, seedling morphology, precipitation seasonality, climate change

## Abstract

**Simple Summary:**

Future precipitation changes are expected to affect plant populations’ adaptive responses. In southern Europe, annual precipitation is expected to decline and become unpredictable with occasional extreme rainfall events. Although there are many studies investigating water deficit effects in beech populations, they mainly refer to water withholding and rewatering or limited watering for prolonged periods. There is a lack of information considering the effect of simulated changes in monthly precipitation distribution on plants. In our study, we aimed to elucidate whether simulated distribution differences in monthly precipitation, expected to prevail in 2050, affects the response of various adaptive traits in beech seedlings originating from sites with contrasting climatic conditions. We found significant population differences according to watering interactions in most of the stem anatomical traits, but only for leaf circularity regarding the morphological traits. Our results indicate that beech populations in the southernmost region of their European distribution may demonstrate high variability in adaptive responses towards climate change conditions.

**Abstract:**

Seasonality, rather than annual precipitation levels, is expected to affect the adaptive responses of plant populations under future climate change. To estimate adaptive traits’ variation, we conducted a common garden experiment with two beech populations from contrasting climatic origins (Evros with longer drought intervals during summer and higher precipitation seasonality, and Drama representing a more temperate ecosystem). We simulated two different watering treatments (frequent vs. non-frequent) on beech seedlings, according to predicted monthly precipitation levels expected to prevail in 2050 by the CSIRO MK3.6 SRESA1B model, considering as reference area a natural beech stand in Mt. Rodopi, Greece. A series of morphological and stem anatomical traits were measured. Seedling survival was greater for the Evros population compared to that of Drama under non-frequent watering, while no difference in survival was detected under frequent watering. Leaf morphological traits were not generally affected by watering frequency except for leaf circularity, which was found to be lower under non-frequent watering for both populations. Stomata density in leaves was found to be higher in the Evros population and lower in the Drama population under non-frequent watering than frequent. Stem anatomical traits were higher under non-frequent watering for Evros but lower for the Drama population. Multivariate analyses clearly discriminated populations under non-frequent rather than frequent watering, indicating genetic adaptation to the population’s environment of origin.

## 1. Introduction

The ongoing climate change is expected to cause prolonged drought periods and changes in seasonal precipitation patterns across the Mediterranean basin, having profound effects on range expansion, adaptive potential and survival dynamics of wild plant populations [1,2]. The above effects can significantly impact the mortality rate of individual plants and cause losses in primary productivity and eventually declines of whole ecosystems [3,4].

One of the plant species that are expected to be mostly affected by climate change is the European beech (*Fagus sylvatica* L.), a dominant forest tree species in Europe that is considered sensitive to extended drought summers [3,5,6,7,8,9,10,11]. Effects of adverse climate conditions connected to climate change on beech forests have been already reported since the beginning of the 21st century [1,12]. During summers with extreme drought events (e.g., 2003 and 2018), most European beech populations were negatively affected [11,13], but the ones growing in Greece were, interestingly, less affected during the summer drought of 2003 [14]. This indicates the existence of intra-specific variation in adaptive traits, defining potential alternative survival strategies among populations growing under different environmental conditions [15,16,17,18,19].

In order to evaluate these different strategies among beech populations, a study of adaptive traits under conditions of water deficit in schemes of common environment experiments is required. Traditionally, such common garden experiments have been utilized to evaluate the performance of forest tree populations of different origins, adapted to different environmental conditions (provenances). In these experiments, the same environmental pressure is applied and different adaptive mechanisms can then be revealed among populations of the same tree species, while genes of adaptive significance can also be identified making this methodology substantial for designing management and conservation plans [20,21]. Additionally, data derived from common garden experiments can be used as base reference for model prediction processes regarding species populations parameters such as growth and distribution under future projected scenarios [22]. 

Plants experiencing prolonged drought intervals between precipitation events usually respond directly to environmental stress by modifying various aspects of their phenology, physiology, morphology and/or anatomy [18,23,24,25,26,27,28,29]. Additionally, plant populations originating from different locations often appear to modify stomata parameters such as size or density, as a result of adaptation to the varying levels of precipitation on their original site [30,31,32].

Plasticity of morphological traits, at the population level, under environmental pressure (e.g., drought) is a common adaptive response [33]. Leaf size and shape are among the most plastic traits; in broadleaved trees, under water deficit, leaf size is reduced and leaves usually become narrower [32,34,35]. As carbon production is reduced under water deficit due to the decrease in photosynthesis rate, plants change their allometric traits such as specific leaf area (SLA), root to shoot ratio and biomass allocation, to adjust growth under the deficit pressure and ensure survival [36,37,38]. Important plastic responses occur in plant anatomical traits as well. Plants under water deficit modify xylem or phloem structural traits, such as conduit diameter or pit membrane characters, or produce more conduits in the same growth period [39,40,41,42,43,44]. 

Apart from the vascular (phloem, xylem) tissues, both ground (cortex, pith) and dermal (phellem) tissues, which provide carbon storage and mechanical support, can change when plants experience a water deficit. Phellem (later named outer bark), which is the main stem protective tissue, usually becomes thicker under water deficit to reduce stem transpiration, maintain hydraulic balance and protect stem photosynthetic activity [45,46,47]. Pith and cortex, which consist of parenchymatous cells that possess storage roles of NSCs in sink organs, while regarding cortex chlorenchyma contributions to the overall carbon fixation through stem photosynthesis, can also be affected by water deficiency [48,49,50,51,52,53].

Common garden experiments investigating the consequences of imposed water deficits on seedlings/saplings of woody plant species usually apply a combination of water withholding followed by rewatering treatments for one or more time periods [10,54,55,56,57,58,59], or continuous water stress (limited watering) for prolonged drought periods, or, also sometimes, water suspension until seedling desiccation [59,60,61,62,63]. However, it is generally accepted that the most striking consequence of climate change is the increased intensity and frequency of extreme weather events [64]. These events are related not necessarily to decreased precipitation, but to shifts in temporal precipitation patterns (e.g., periods of extended drought followed by heavy rainfall events). Common garden experiments simulating this kind of extreme weather events, based on future projected climatic conditions, to study plant adaptive traits are generally lacking and are highly essential to the good practice of forest management. 

In our study, we established a six-year common garden experiment where beech seedlings originating from two populations growing under contrasting environmental field conditions were subjected to simulated irrigation frequency treatments according to the monthly precipitation levels expected to prevail in 2050. We aimed to assess whether the tested beech provenances would respond differently under different precipitation frequencies regarding anatomical and morphological traits. We expected the beech population originating from an area experiencing increased summer drought to have a higher adaptive capacity to extreme precipitation patterns. 

## 2. Materials and Methods

### 2.1. Selected Populations and Plant Material

**Figure 1:**
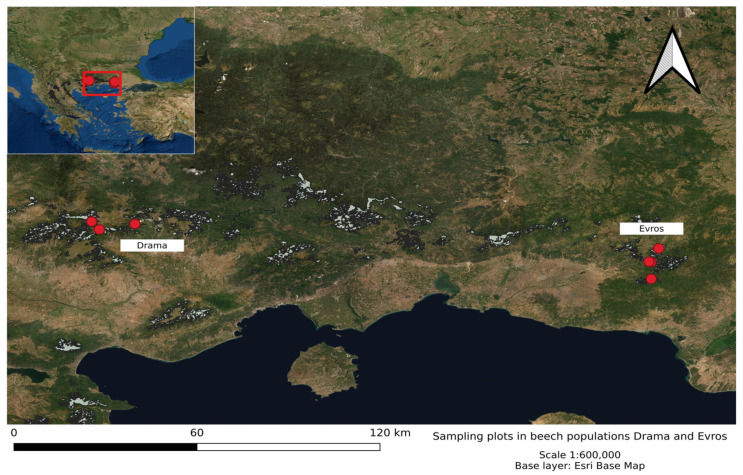
Map with the location of sampling plots in the beech populations Drama and Evros in NE Greece. Light-gray shading indicates the area covered by beech forests in Greece.

Two natural beech populations growing in locations with different climatic conditions in northeastern Greece, Evros and Drama, were selected for this study (Figure 1). Evros lies on the eastern side of the Greek part of the Rodopi mountain range and is characterized by a Meso-Mediterranean (attenuated) climate type with cold and moist winters, but warm summer periods of increasing drought duration. Drama is located on the western edge of the Rodopi mountains and resembles a Sub-Mediterranean climate type with high continentality, severe winters and generally less warm summers with a smaller duration of droughts. Bioclimatic factors for the selected populations are displayed in Table S1. Although Evros receives higher levels of annual precipitation compared to Drama, Evros demonstrates higher precipitation seasonality. More specifically, precipitation of the coldest quarter is higher in Evros as compared to Drama. This shows that Evros receives most of its annual precipitation during winter. At the same time, precipitation of both the driest month and the warmest quarter is lower in Evros. Finally, the drought period lasts longer in Evros and the index is higher there in contrast to Drama (Table S1). These beech populations (Drama and Evros) were included in previous genetic studies using neutral molecular markers and were found to differ [65,66]. Most interestingly, comparisons between the same two beech populations in common garden experiments have revealed significant differences in adaptive traits of seedlings in bud burst phenology [18] and seed germination attributes [19]. 

Within each population, four representative sampling plots were selected, but finally one plot from Drama was excluded from the trial due to the abnormal development of the produced seedlings, according to ISTA rules [67]. Within each plot (a total of four plots from Evros and three plots from Drama), seeds were collected from 30 individual trees, in October 2012. Collected seeds were cleaned, subjected to cold moist stratification at 0 °C for 90 days and finally germinated under alternate temperatures (+25 °C/+15 °C) and photoperiod conditions (8 h/16 h, light/dark). Germinants were initially planted in pots filled with Sand:Turf:Perlite at 4:2:1 ratios, respectively. The produced seedlings were monitored for one month and those that developed abnormally were discarded [67]. 

### 2.2. Experimental Design and Simulated Climate Change Precipitation Schemes

The effect of future climate change conditions on adaptive traits of beech seedlings was investigated through a six-year simulation experiment established in two plant growth chambers. The reference area for the simulation of the climate conditions was a natural beech forest located in the Drama region (41°17′29.47″ N, 23°55′17.69″ E). Future monthly temperature and precipitation data for the year 2050 under the CSIRO MK3.6 (SRES A1B) model were downloaded from “climond.org” database in the .GRD format and processed to produce a spreadsheet with these variables on a monthly basis [68,69] (Table S2). Seedlings were grown under a common temperature scheme and two different watering treatments (frequent watering (FR): watering every 7 days; and non-frequent watering (NF): watering every 20 days) simulating different monthly precipitation patterns. The amount of watering for each month of the experiment was the same for both watering treatments and corresponded to the estimated rainfall of the specific month under the CSIRO MK 3.6 and SRES A1B. The simulated experimental design is also described in full detail in Varsamis et al. [18]. The experiment ended in October 2019 and the seedlings were subjected to the destructive measures described below. The seedlings’ final survival was calculated on a percentage basis.

### 2.3. Morphological and Anatomical Traits

Three fully developed leaves per seedling were collected in July 2019 and digitally scanned on a portable hp Scanjet 5590 flatbed scanner device (HP Development Company, L.P.). The area of the lamina was measured alongside with additional morphological traits using Image J v. 1.50i (http://imagej.nih.gov/ij, accessed on 12 January 2022, USA). The traits measured were the angle between leaf tip and base, the number of secondary veins, leaf length and width as well as leaf circularity (Table 1). In the middle of the abaxial lamina, stomatal density was measured using the nail varnish imprinting method [70]. Three neighboring images per leaf slide were captured under 250× magnification on a Nikon microscope, the mean stomatal number per mm^2^ leaf area was calculated for every leaf and then the mean stomatal number of the three leaves was further calculated [62,70]. Then, individual leaf dry weight was determined after oven-drying at 70 °C for 24 h [71]. Specific leaf area was calculated as the ratio of the leaf area to the dry weight, to describe the balance of carbon acquisition and use [72]. Finally, leaf thickness was indirectly estimated by calculating specific leaf area and leaf dry matter content following Vile et al. [73]. 

In October 2019, after the current growth period had ended, the seedlings were removed from the pots and separated into their shoot and root parts by cutting the seedlings with a pair of scissors at the root collar. Shoot length was measured with a ruler, while the root (including primary, seminal and lateral roots) was scanned on a flatbed scanner device and the root surface area was measured using Image J. Then, both shoot and root dry weights were measured after oven-drying at 70 °C for 24 h. Prior to oven-drying, a 1 cm shoot piece was cut, fixed in a mixed solution of 80% ethanol (100% stock solution) and 20% glycerol (70% stock solution) that was renewed daily for 5 days and subjected to sectioning at a cryotome at 30 μm (modified protocol from Jupa et al. [74]). The sections were then mounted to a glass slide at a thermoplate at 37 °C for 20 min using Haupt’s adhesive recipe [75]. Afterwards, they were immersed in 0.1% toluidine blue for two minutes, washed with distilled water and dehydrated in ascending ethanol series [76]. Finally, in each section in the glass slide, a drop of 70% glycerol and a coverslip were added. Sections were examined under 100× magnification on a microscope. The measured stem adaptive traits were: the section area, the length of phellem, cortex, phloem and xylem, and the pith length alongside the number of pith rays [77] (Table 1). 

### 2.4. Statistical Analyses 

Mean trait values were calculated for all traits at the population level. Normality was checked with the Kolmogorov–Smirnov test and all traits had values following the normal distribution, apart from the number of pith rays, the root area, the number of secondary leaf veins and the leaf length. The normality of the latter traits was corrected by using arcsine transformation. Differences in trait mean values were checked with an independent *t*-test for all variables except for the number of secondary leaf veins, where the Mann–Whitney test was used.

A principal component analysis (PCA) using the “FactoMineR” package of R [78] was performed separately for each of the watering treatments. Finally, mixed model analysis was performed using the SPSS v.19 software (SPSS Inc., Chicago, IL, USA) considering individual seedlings as a random factor and watering treatment and population as fixed factors. The maximum likelihood was used as an estimator of model parameters.

## 3. Results

### 3.1. Seedling Survival

**Figure 2:**
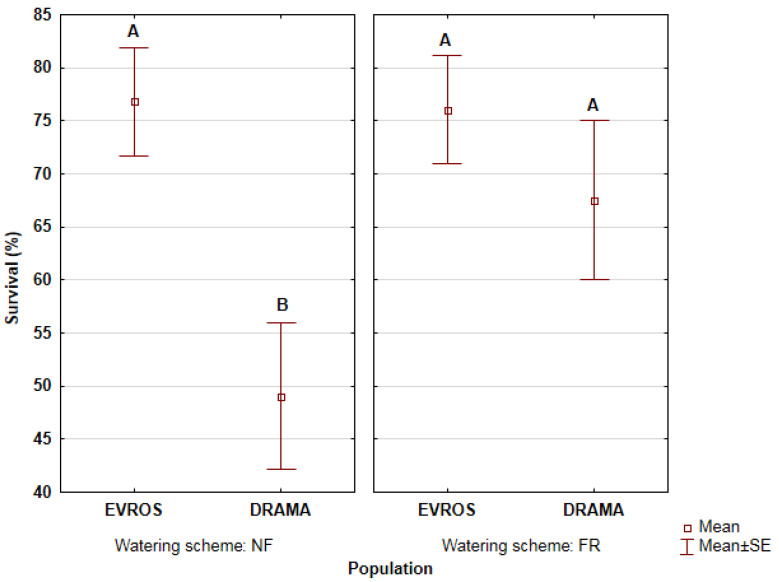
Survival percentages of beech seedlings at the end of the experiment under each watering treatment. The vertical lines show the standard error of mean values. Mean values between populations followed by the same capital letter (separately for each watering treatment) do not statistically differ at 5% level of significance.

The percentage of survived seedlings differed between populations under non-frequent watering. Seedlings originating from Evros presented significantly higher survival than those from Drama (Figure 2). On the other hand, no significant differences between populations were observed under frequent watering.

### 3.2. Differences between Watering Treatments

Among all traits, leaf circularity and phloem length were the only traits found to differ significantly between non-frequent and frequent watering treatments (Table 2). However, when the interaction between population and treatment was considered, different trends in changes between treatments were described at the level of each population (Table 2 and Table S3). Both in Evros and Drama, leaf circularity values were higher under frequent watering, but this difference was greater in Drama. Additionally, only the seedlings of Evros presented higher root and shoot dry weight under non-frequent watering. Regarding stem anatomical traits, seedlings from Evros presented higher values under non-frequent watering, except from the number of pith rays, where no difference between the watering treatments emerged. In Drama, seedlings under non-frequent watering presented lower section area, phloem and xylem length, but higher stomatal number.

### 3.3. Differences between Populations

Mixed model analysis revealed significant differences in most of the measured traits between the studied populations. Among the leaf morphological traits and under non-frequent watering, leaf circularity and leaf length were significantly different between the populations (Table 2), while no significant differences were found at any of the leaf morphological traits under frequent watering. In Evros, leaf circularity was higher and leaf length received lower values under non-frequent watering, in comparison to Drama (Table 2). 

Shoot length presented a marginal differentiation between populations, independent of the applied watering treatment, with Drama having higher values than Evros. Regarding stem anatomical traits, under non-frequent watering, Evros had lower leaf stomatal density, with thicker stem (i.e., higher section area) and higher cortex, phloem, xylem length and pith length than Drama. On the contrary, phellem length and the number of pith rays were the only anatomical traits showing no significant differentiation between populations under non-frequent watering, but they significantly differed under frequent watering. Phloem and xylem length were significantly different between populations at both non-frequent and frequent watering treatments. Under frequent watering, phloem, xylem and phellem length showed higher values in seedlings from Drama, while the number of pith rays was higher in Evros. 

**Figure 3:**
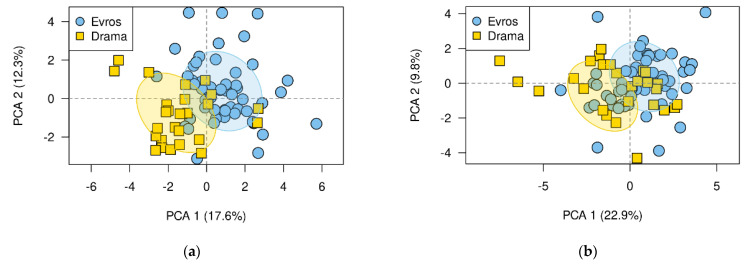
PCA plot of individuals, under (**a**) non-frequent and (**b**) frequent watering.

Furthermore, the results of the mixed model analysis showed a significant “population x watering treatment” interaction only for leaf circularity and most of the stem anatomical traits (i.e., section area, phloem length, xylem length, pith length and stomatal density) (Table 2). Finally, the PCA plot of individual trees clearly separated the Drama and Evros populations from each other under non-frequent watering, but not under frequent watering (Figure 3).

## 4. Discussion

### 4.1. Seedling Response to Watering Frequency

Our results demonstrate that watering frequency is an important parameter for beech seedling survival, even when the total amount of water provided remains constant. Non-frequent watering seems to trigger phenotypic changes, especially in seedling stem and root anatomical traits, but this response was different across the studied beech populations. 

Seedling survival at the end of the experiment was significantly affected by watering frequency. Survival percentage was lower for Drama seedlings under non-frequent than frequent watering, while in Evros survival did not differ between frequent and non-frequent watering. Thus, Evros proved to be more adapted to longer drought intervals than Drama and this could be attributed to adaptation to the environmental conditions of its site of origin, where precipitation seasonality and summer aridity are higher. Ιt seems that even though the differences in precipitation of the driest quarter between Evros and Drama are small, they are still decisive for shaping seedling survival. 

The only trait affected in a similar way for both populations was leaf circularity, while no significant changes due to the different watering treatments were recorded for the other leaf morphological traits. Non-frequent watering caused a significant decrease in leaf circularity, which indicates that seedlings reacted to long periods of drought by changing their leaf shape. Similarly, Xu et al. [79] report that the leaves of *Quercus acutissima* became narrower under drought because of limited watering, and a similar trend was reported by Granda et al. [35] in clones of *Eucalyptus globulus*. 

A possible explanation for the overall lack of significant differences in beech leaf morphology under different watering frequencies may be a possible thermal stress that seedlings may have experienced during the experiment. The photosynthetic apparatus of beech is reported to be highly sensitive to thermal stress [11,80], leading to the reduction of the available leaf surface to reduce photosynthetic activity of the plants, as a response [81,82,83,84,85]. Thus, we can assume that under a potential thermal stress under the controlled environment, the leaves became smaller and no further leaf size reduction was required for the seedlings to meet limitations by non-frequent watering. 

The two beech populations studied, Evros and Drama, demonstrated contrasting seedling responses to different watering frequency treatments, at important adaptive traits. These traits include stomatal number in leaves and stem anatomical traits. When watering occurred less frequently, seedlings belonging to the Evros population had a significantly lower density of stomata on their leaves, which is a typical response of beech populations when transferred to drier and warmer climates [32]. The same treatment caused the opposite trend in seedlings from Drama, which thus had increased stomatal density on their leaves when watering occurred less frequently. This opposite response of Drama seedlings can be the result of adaptation to the prevailing precipitation pattern at their original environment during leaf development (Figure S1). Between spring and early summer, when leaves are growing, the monthly precipitation course follows a relatively uniform pattern, in contrast to Evros where it follows a continuous reduction until the end of summer. 

A similar contrasting pattern between the two populations was observed when the anatomical traits for the stem vascular system and the overall structure of shoots and roots of the seedlings were compared for the two watering treatments. Non-frequent watering caused an increase in xylem, phloem, cortex, pith and phellem length among Evros seedlings, while the opposite trend was found in Drama. Thus, Evros seedlings produced more annual xylem and phloem under prolonged intervals between watering events resulting in a more robust vascular system with thicker xylem and phloem tissues, which possibly offers better protection against embolism and loss of cell turgor [28,42,86]. For the above protection path, the higher cortex can also contribute since it serves as sink tissue for low viscosity. The overall increase in stem thickness (higher section area) may also provide a more efficient water storage capacity for the seedlings [74]. The opposite was observed in seedlings belonging to Drama, where stem thickness was reduced, a response that was observed as a typical reaction of beech seedlings to drought [87,88]. 

Subsequently, root and shoot dry weight of beech seedlings from Evros were found to be significantly greater under non-frequent irrigation than under the frequent watering treatment. Despite the fact that water deficit is expected to generally cause biomass reduction to beech seedlings [87,89] and to root biomass in seedlings of the same tree species [58], the seedlings from Evros in our study demonstrated increased resource allocation in sink tissues under non-frequent watering. A possible explanation for this trend is the fact that the non-frequent watering treatment is not equivalent with the usually applied increased drought treatments. Additionally, biomass allocation to both root and shoot parts increases usually at mid-summer when precipitation is more scarce. It seems possible that the Evros seedlings, originating from a site where summer precipitation is less frequent and probably more unpredictable, better tolerated the higher watering interval, resulting in increased shoot and root biomass allocation. By contrast, seedlings from Drama followed the expected response against drought, thus demonstrating a moderate biomass reduction in their shoot and root tissues under the non-frequent watering treatment.

Furthermore, the root-to-shoot ratio was not affected in our case by the different watering treatments in each population, which agrees with other common environment studies for beech [24,89] and for *Quercus robur* [90]. However, Rose et al. [23] found a significant effect of water deficit on root/shoot ratio for beech populations in a common garden experiment. 

### 4.2. Adaptive Differences between Populations

Under the simulated conditions of the year 2050 in the growth chamber, beech seedlings from Drama and Evros demonstrated differences in important adaptive traits. However, the only traits for which significant differences between the populations were observed, regardless of the watering treatment applied, were leaf size and shoot length. Drama seedlings had larger leaves and longer shoots than the ones from Evros. For most of the traits, and especially for the anatomical ones, the differences between the two populations followed different trends under the applied watering treatments, as explained above. Thus, the most noteworthy result of this experiment is the interaction between watering treatment and population, especially as far as the density of the leaf stomata, the stem anatomy and the overall biomass accumulated in shoots and roots is concerned.

These results strongly indicate that these two beech populations have adaptive differences towards the occurrence of longer drought periods caused by seasonal irregularities in precipitation. Despite receiving the same monthly amount of water, the distribution of monthly precipitation appears to have played a major role in the strategy followed by the seedlings of each population. Under less frequent water supply, seedlings from Drama seem to follow a conservative growth pattern by reducing their vascular area and decreasing stem growth, a typical drought avoidance reaction for temperate broadleaved trees [91,92]. Under the same conditions, seedlings from Evros seemingly invest more in better supporting their vascular system and acquire higher amounts of water and biomass gains when the resources are available, demonstrating an alternative strategy for coping with long drought intervals. In a review by Geßler et al. [1] it is reported that seasonal precipitation irregularities affect the growth of beech populations, while water-logged soils during spring for several regions due to higher-than-average precipitation could negatively impact the nutrient uptake and growth of beech. Thus, fewer but intense precipitations are potentially a stress factor for beech seedlings from Drama, but they seem to provide an opportunity for increased growth for beech seedlings from Evros. 

## 5. Conclusions

The future of beech populations in the southern part of its distribution in Europe is uncertain, due to the climatic fluctuations that are expected to become more extreme in the next decades [93]. Pflug et al. [26], Gebauer et al. [58] and Larysch et al. [94] found high resilience of beech seedlings under spring drought, but they considered this resilience to be possibly weakened under repeated intense drought events, especially in the southernmost distributional limits of beech in Europe. However, beech populations on the southeastern part of Europe have demonstrated large genetic diversity [95,96] and significant differentiation in adaptive traits [18,19]. The latter is further supported by our results, where seedlings seemed to cope with the effects of prolonged intervals between irrigation, following different adaptive strategies. While the seedlings from Evros showed a better survival under the 2050 conditions with longer drought intervals during summer, both populations demonstrated different strategies of plastic responses to these conditions, which have allowed the seedlings to finally survive for several years. Furthermore, these differences were evident under simulated climatic instability, with scarce, irregular and intense precipitation events, conditions that are expected to increase dramatically in the near future. 

Our results indicate the existence of high adaptive potential as well as differentiation among populations in the rear edge of the beech distribution in Europe and demonstrate the importance of these populations for the future survival of beech in the continent, especially as far as management and conservation policies are concerned [97,98,99]. Populations of temperate species in the southern part of Europe are expected to suffer most under climate change, but they also demonstrate adaptive differences and high genetic diversity. Thus, these populations may become the basis for future common garden experiments further north, in regions where translocation of forest seed material is discussed as a measure to enrich genetic diversity locally and cope with the expected dramatic changes in climatic conditions in the continent. Understanding the adaptation mechanisms of plant populations from broader geographic regions is extremely important before making decisions about measures for conservation and management of tree genetic resources. 

## Figures and Tables

**Table 1:** Definition of the measured traits.

Trait	Description
Specific leaf area (SLA)	The ratio of leaf area to dry weight.
Leaf dry matter content (LDMC)	The ratio of leaf fresh weight to its dry weight.
Leaf thickness	The estimated leaf lamina thickness.
Leaf length	The length of leaf lamina.
Leaf width	The width of leaf lamina at its maximum.
Leaf base angle	The angle of leaf lamina base.
Leaf tip angle	The angle of leaf lamina tip.
Number of leaf secondary veins	Number of first-class veins.
Leaf circularity	The ratio of area to perimeter of leaf.
Shoot length	The length from the root collar to the apical bud.
Shoot dry weight	The shoot weight after drying in an oven.
Root area	The projected area of the whole seedling root system.
Root dry weight	The root system weight after drying in an oven.
Section area	Total projected area of the produced stem section.
Phellem length	Length of the outer plant suberized epidermis.
Cortex length	Length of the tissue layer between epidermis and phloem tissue.
Phloem length	Length of the tissue layer between cortex and xylem.
Xylem length	Length of the tissue layer between phloem and pith.
Pith length	Maximum length of the pith
Stomatal density	Density of stomata number in the middle leaf lamina part.
Number of pith rays	The number of the rays that connect the vascular system (xylem, phloem) with the pith.

**Table 2:** Results of mixed model analysis for trait differentiations across watering treatments and populations.

Trait	Factor		
	Population	Watering Treatment	Population × Watering Treatment
Specific leaf area	0.315	0.133	0.806
Leaf dry matter content	0.757	0.216	0.146
Leaf thickness	0.811	0.449	0.375
Leaf length	0.002 *	0.262	0.518
Leaf width	0.005 *	0.333	0.552
Leaf base angle	0.517	0.716	0.153
Leaf tip angle	0.083	0.801	0.454
Number of secondary leaf veins	0.715	0.016	0.250
Leaf circularity	0.025 *	0.000 ***	0.001 ***
Shoot length	0.005 **	0.200	0.601
Shoot dry weight	0.142	0.342	0.200
Root area	0.166	0.677	0.284
Root dry weight	0.535	0.357	0.110
Section area	0.000 ***	0.559	0.000 ***
Phellem length	0.104	0.752	0.106
Cortex length	0.727	0.465	0.001 ***
Phloem length	0.064	0.040 *	0.000 ***
Xylem length	0.656	0.087	0.000 ***
Pith length	0.002 **	0.214	0.000 ***
Stomatal number	0.001 ***	0.521	0.008 **
Number of pith rays	0.001	0.682	0.430

Bold numbers represent significant differentiation (***, *p* < 0.001; **, *p* < 0.01; *, *p* < 0.05).

## Data Availability

All data included in this study are available from corresponding authors, upon request.

## Supplementary Materials

The following are available online at https://www.mdpi.com/article/10.3390/biology11020306/s1, Figure S1: Precipitation at the original sites of the studied populations; Table S1: Bioclimatic variables at the original sites of the studied populations (downloaded and extracted from www.worldclim.org, accessed on 16 December 2021); Table S2: Values of climatic variables for study populations under CSIRO MK3.6 SRES A1B for the year 2050 (downloaded and extracted from www.climond.org, last accessed: 13 February 2013). Table S3: Descriptive statistics (mean value ± standard error) of beech provenances under the applied watering treatments.
