# Supplementary material for: Changes in Watering Frequency Stimulate Differentiated Adaptive Responses among Seedlings of Different Beech Populations
Source: Biology (Basel). 2022 Feb 14;11(2):306. doi: 10.3390/biology11020306 (PMC8868575; doi:10.3390/biology11020306)
Supplement: Supplementary file 1 [file biology-11-00306-s001.zip › Table S3.pdf]

Table S3. Descriptive statistics (mean value ± standard error) of beech provenances under the applied watering treatments

Trait	Evros		Drama	
	FR	NF	FR	NF
	Mean value	Mean value	Mean value	Mean value
Specific leaf area-SLA ($mm^2/\mu g$)	325.582±12.344	348.505±11.962	315.632±8.005	332.130±7.867
Leaf dry matter content-LDMC	1.347±0.019	1.253±0.028	1.281±0.025	1.202±0.024
Leaf thickness (μm)	0.270±0.013	0.249±0.010	0.262±0.009	0.264±0.008
Leaf length (mm)	33.255±1.034	30.956±0.933	36.478±1.414	35.860±1.012
Leaf width (mm)	21.076±0.656	20.781±0.597	23.783±0.807	22.545±0.589
Leaf base angle (°)	106.556±1.670	110.264±1.838	108.168±1.383	105.967±1.851
Leaf tip angle (°)	91.381±1.007	92.839±1.457	89.947±1.333	89.223±0.863
Number of leaf secondary veins	15.056±0.365	14.537±0.344	15.679±0.272	14.214±0.348
Leaf circularity	0.737±0.007	0.719±0.006	0.746±0.005	0.677±0.006
Shoot length (cm)	14.009±0.407	14.912±0.378	15.665±0.424	16.044±0.478
Shoot dry weight (mg)	0.273±0.014	0.334±0.014	0.344±0.022	0.318±0.011
Root area (mm^2)	1187.871±103.726	1440.010±197.157	1134.648±116.171	1023.683±83.732
Root dry weight (mg)	0.317±0.025	0.389±0.022	0.380±0.028	0.361±0.020
Section area (μm^2)	2338.964±94.700	2991.380±105.960	2528.972±147.617	1729.094±55.288
Phellem length (μm)	19.152±0.723	20.537±0.497	21.477±0.479	20.544±0.594
Cortex length (μm)	39.298±1.567	46.935±1.231	44.848±2.208	40.035±1.557
Phloem length (μm)	66.815±2.279	87.183±2.772	76.625±1.859	67.349±1.126
Xylem length (μm)	374.464±11.996	501.306±18.693	461.783±14.296	397.644±14.144
Pith length (μm)	470.555±15.176	552.099±14.212	479.867±13.107	439.907±11.140
Stomatal density	127.247±3.041	117.854±3.302	129.857±5.208	145.298±4.752
Number of pith rays	19.333±0.482	19.130±0.409	17.179±0.279	17.821±0.557
