# Supplementary figures and images for: Changes in Watering Frequency Stimulate Differentiated Adaptive Responses among Seedlings of Different Beech Populations
Source: Biology (Basel). 2022 Feb 14;11(2):306. doi: 10.3390/biology11020306 (PMC8868575; doi:10.3390/biology11020306)

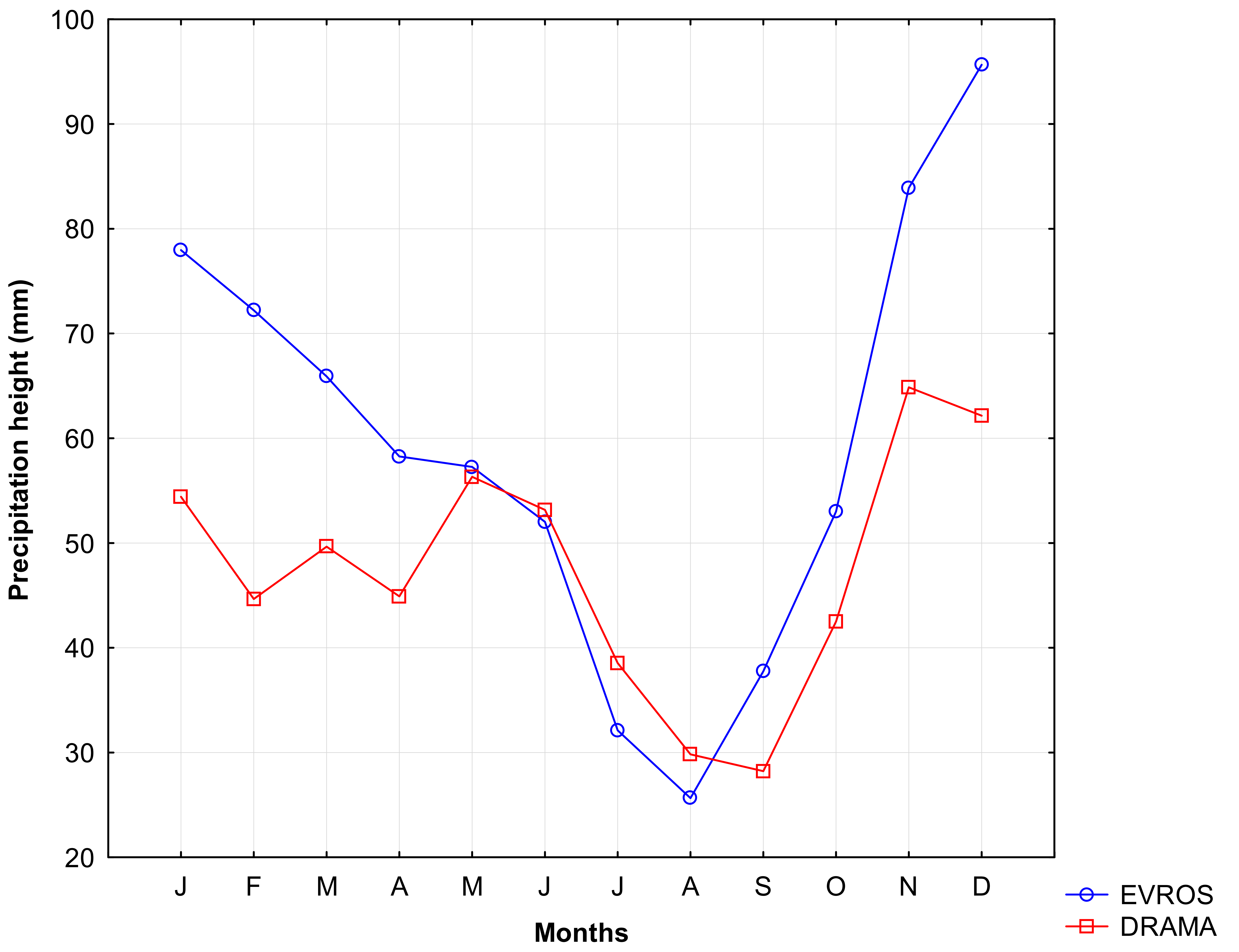

Supplement: Supplementary file 1 [file biology-11-00306-s001.zip › Figure S1.tif]
